# Proteomic characterization of hUC-MSC extracellular vesicles and evaluation of its therapeutic potential to treat Alzheimer’s disease

**DOI:** 10.1038/s41598-024-56549-6

**Published:** 2024-03-12

**Authors:** Shuang Li, Jiayi Zhang, Xinxing Liu, Ningmei Wang, Luyao Sun, Jianling Liu, Xingliang Liu, Abolfazl Masoudi, Hui Wang, Chunxia Li, Chunyan Guo, Xifu Liu

**Affiliations:** 1 Ministry of Education Key Laboratory of Molecular and Cellular Biology, Hebei Anti-Tumour Molecular Target Technology Innovation Center, College of Life Science, Hebei Normal University, Shijiazhuang, 050024 China; 2 Jianyuan Precision Medicines (Zhangjiakou) Co., Ltd., Zhangjiakou, 075000 China; 3 Hebei Key Laboratory of Neuropharmacology; Department of Pharmacy, Hebei North University, Zhangjiakou, 075000 China; 4 Cancer Research Institute, The First Affiliated Hospital of Hebei North University, Zhangjiakou, 075000 China; 5 Department of Neurology, The First Affiliated Hospital of Hebei North University, Zhangjiakou, 075000 China; 6 Obstetrics and Gynaecology, The Fifth Hospital of Zhangjiakou, Zhangjiakou, 075000 China

**Keywords:** LC‒MS/MS, hUC-MSC-EVs, Proteomics, Alzheimer's disease, Cell biology, Neuroscience, Stem cells

## Abstract

In recent years, human umbilical cord mesenchymal stem cell (hUC-MSC) extracellular vesicles (EVs) have been used as a cell replacement therapy and have been shown to effectively overcome some of the disadvantages of cell therapy. However, the specific mechanism of action of EVs is still unclear, and there is no appropriate system for characterizing the differences in the molecular active substances of EVs produced by cells in different physiological states. We used a data-independent acquisition (DIA) quantitative proteomics method to identify and quantify the protein composition of two generations EVs from three different donors and analysed the function and possible mechanism of action of the proteins in EVs of hUC-MSCs via bioinformatics. By comparative proteomic analysis, we characterized the different passages EVs. Furthermore, we found that adaptor-related protein complex 2 subunit alpha 1 (AP2A1) and adaptor-related protein complex 2 subunit beta 1 (AP2B1) in hUC-MSC-derived EVs may play a significant role in the treatment of Alzheimer's disease (AD) by regulating the synaptic vesicle cycle signalling pathway. Our work provides a direction for batch-to-batch quality control of hUC-MSC-derived EVs and their application in AD treatment.

## Introduction

Human umbilical cord mesenchymal stem cells (hUC-MSCs) exhibit low immunogenicity and multilineage differentiation. They have promising therapeutic effects including promoting tissue repair and regeneration and thus are ideal seed cells for transplantation^1–3^. However, after mesenchymal stem cells (MSCs) are implanted into the body, there can be several side effects. Uncontrollable immune regulation, abnormal accumulation, and nontherapeutic differentiation have limited the clinical application of hUC-MSCs to some extent^4–6^. An increasing number of studies have suggested that hUC-MSCs secrete a variety of cytokines through a paracrine mechanism to activate tissue repair^7,8^. Histological data also show that hUC-MSCs can secrete a variety of bioactive proteins during growth and expansion^9,10^. Therefore, the paracrine bioactivity of MSCs has garnered much attention in both academic and clinical research.

EVs are spherical particles released from cells by paracrine signalling after the fusion of polyvesicles and cell membranes^11^. Previous studies have shown that human umbilical cord mesenchymal stem cell-derived extracellular vesicles (hUC-MSC-EVs) can regulate the activity of target cells by transferring proteins to target cells^12^. HUC-MSC-EVs have been shown to play a therapeutic role in various injury types and diseases, including liver injury, spinal cord injury, autoimmune disease, myocarditis, skin injury, and peripheral nerve injury^13–18^. Compared with those used in cell therapy, EVs are more stable and convenient for preservation and transportation and are therefore expected to become new biological agents. According to the latest data from ClinicalTrials.gov (https://clinicaltrials.gov), there have been about 30 clinical studies of EVs in treating diseases, but so far no relevant drug has been approved for marketing. The launch of EVs’ drug still have challenges in developing specific potency assays and ascertaining the life span.

**Figure 1:**
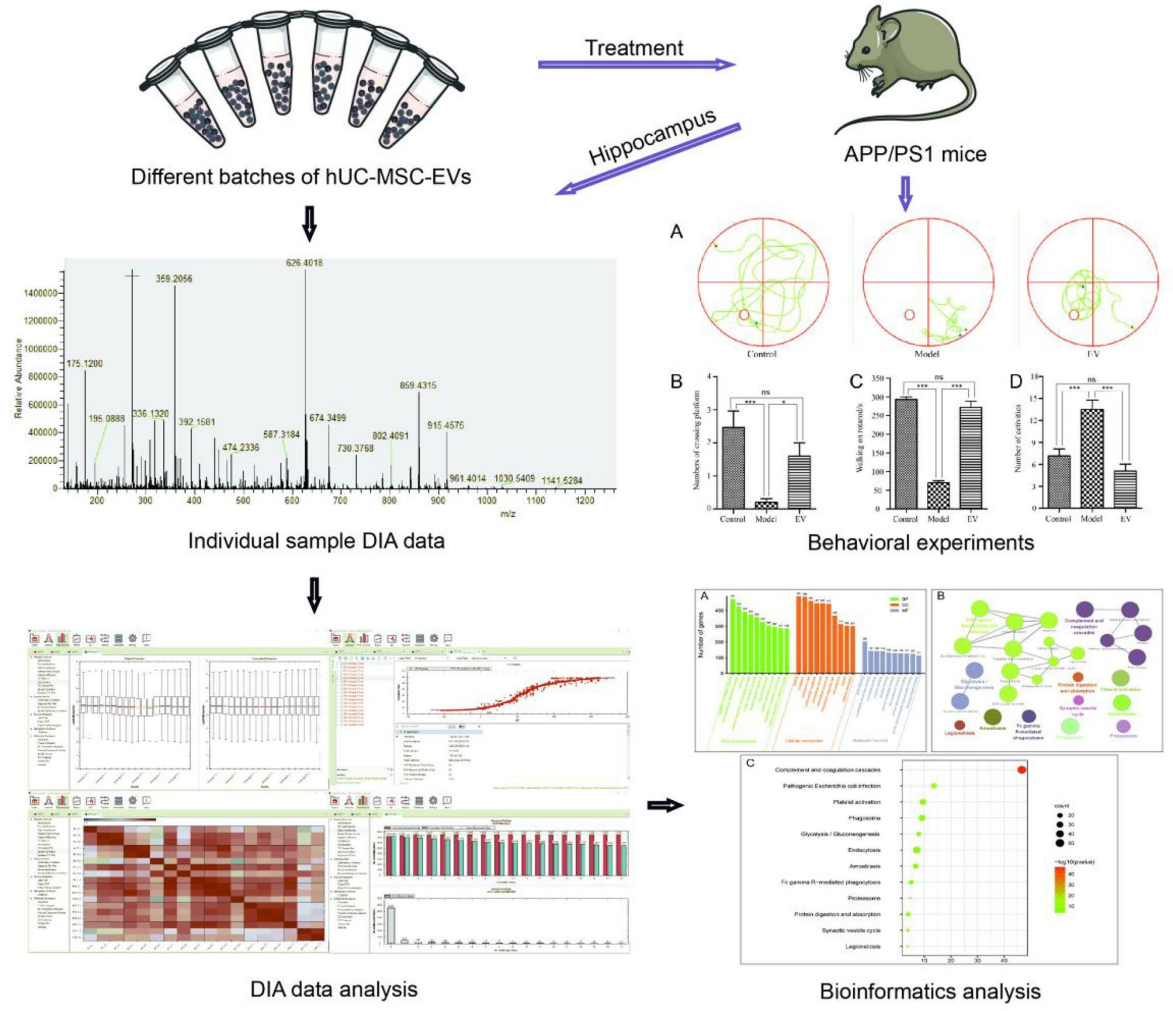
Workflow of DIA-based quantitative proteomic analysis of hUC-MSC-EVs and disease intervention. Six batches of hUC-MSC-EV proteins were extracted by enzymatic hydrolysis, and the data were collected by a Q Exactive HF mass spectrometer through the quantitative DIA protein method. The protein spectra were analysed, and a variety of bioinformatics methods were used to analyse the function and possible mechanism of the hUC-MSC-EV proteome. The therapeutic efficacy and mechanism of action in treating nervous system diseases were verified by treating APP/PS1 mice with hUC-MSC-EVs.

To date, several studies have investigated the composition and partial functions of the mRNAs and proteins in hUC-MSC-EVs^9,19–22^. However, little is known about the specific mechanism of hUC-MSC-EV intervention in AD treatment, and whether there are differences in protein composition among different donors and generations has not been determined. In this study, EVs extracted from two generations of hUC-MSCs from three different donors were used. The composition of proteins in the EVs of the six groups was determined by DIA-based quantitative proteomics^23^ and the function and possible mechanism of the hUC-MSC-EV proteome were analysed via bioinformatics. The therapeutic potential and possible mechanism of action of the hUC-MSC-EVs used to treat AD were investigated and verified by hUC-MSC-EV intervention in APP/PS1 mice, a mouse model of AD. The overall flow of the experimental scheme is shown in Fig. 1. These research results can provide a theoretical basis for the use of hUC-MSC-EVs in the treatment of nervous system diseases and further promote the clinical application of hUC-MSC-EVs.

## Results

### Biological characteristics of hUC-MSCs

**Figure 2:**
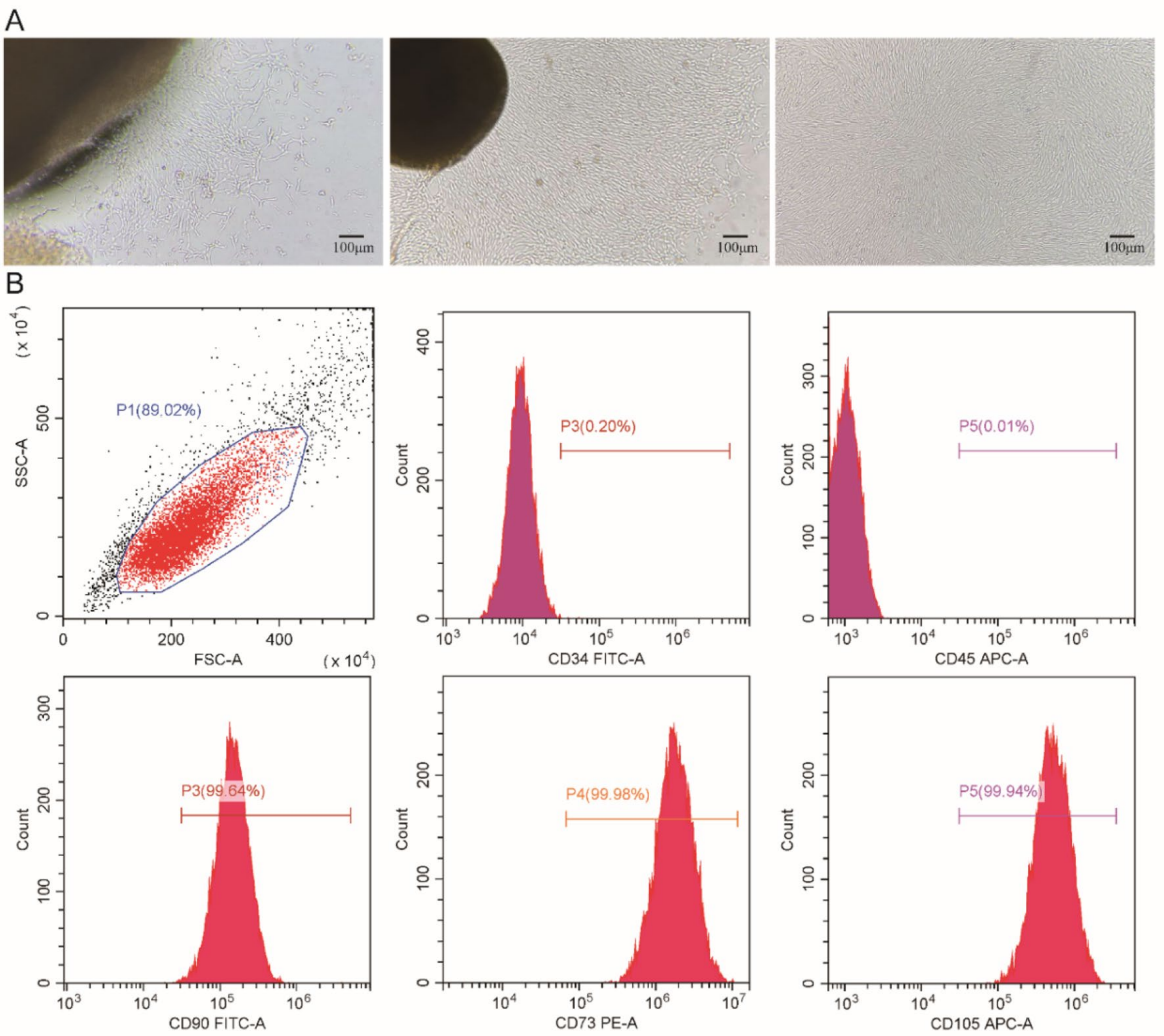
Growth and biological characteristics of hUC-MSCs. (**A**) hUC-MSC growth and morphology were observed under an inverted microscope (× 4). The left panel shows a few adherent cells on day 3 of culture. The middle graph shows that many cells migrated after 5–9 days of culture. The figure on the right shows that the cell morphology at passage 4 was uniform and the cells had a short spindle shape with vortex growth. (**B**) Flow cytometry was used to analyse cell surface antigens of human umbilical cord mesenchymal stem cells. In the figure, from left to right, are the column diagrams of the SSC-FSC double-scattered light parameters, FITC-CD34, APC-CD45, FITC-CD90, PE-CD73, and APC-CD105.

After stable passaging, cultured hUC-MSCs showed a uniform short spindle shape that adhered to the wall under the microscope, consistent with the required morphology of hUC-MSCs (Fig. 2A). Flow cytometry revealed (Fig. 2B) that these cells did not express the haematopoietic stem cell surface marker CD34+ (0.20%) or the leukocyte surface marker CD45+ (0.01%). However, there was CD90+ (99.64%), CD73+ (99.98%), and CD105+ (99.94%) expression. The above results indicated that the cells cultured in this study were at high-purity of hUC-MSCs.

### Identification of hUC-MSC-EVs

**Figure 3:**
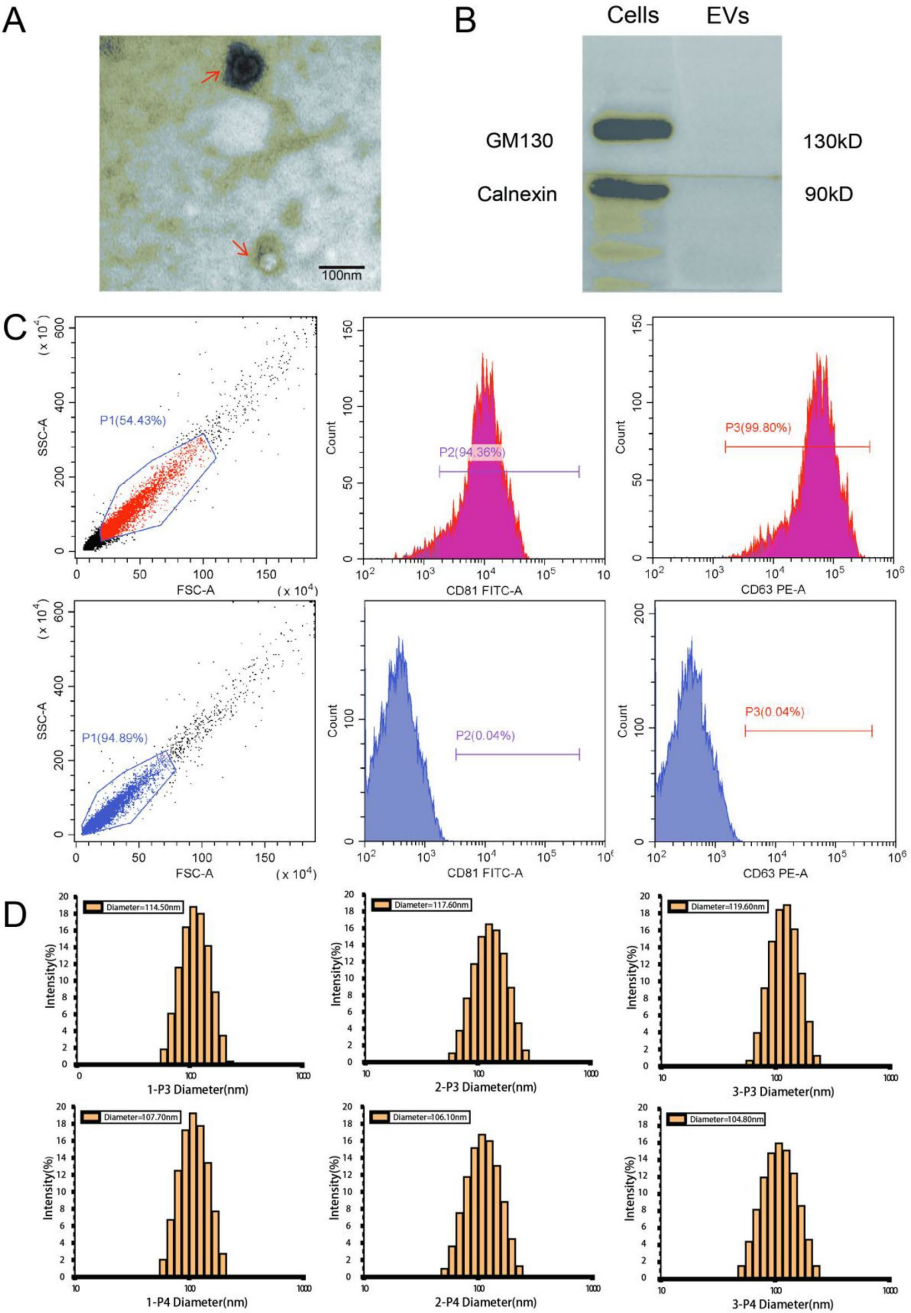
Identification of hUC-MSC-EVs. (**A**) The extracted hUC-MSC-EVs exhibited an irregular circular shape under TEM. (**B**) Western blot analysis of the expression of GM130 and calnexin in cells and in EVs was performed. (**C**) Flow cytometry results show that the extracted hUC-MSC-EVs expressed CD81 and CD63. In the figure, from left to right, are the column diagrams of the SSC-FSC double-scattered light parameters, FITC-CD81, and PE-CD63. The three negative controls are EVs that were not fluorescently stained. (**D**) The NTA plot shows the diameter of isolated EVs.

Transmission electron microscopy (TEM) confirmed that the extracted hUC-MSC-EVs were irregular and circular (Fig. 3A). Flow cytometry and Western blot indicated that the EVs were positive for the markers CD81 and CD63, but negative for the markers GM130 and calnexin as negative controls (Fig. 3B,C). A nanoparticle tracking analyser (NTA) revealed that the average particle size was approximately 100 nm (Fig. 3D). These characteristics confirmed the presence of hUC-MSC-EVs in our extract.

### Evaluation of the proteome of hUC-MSC-EVs from different donors and culture generations

**Figure 4:**
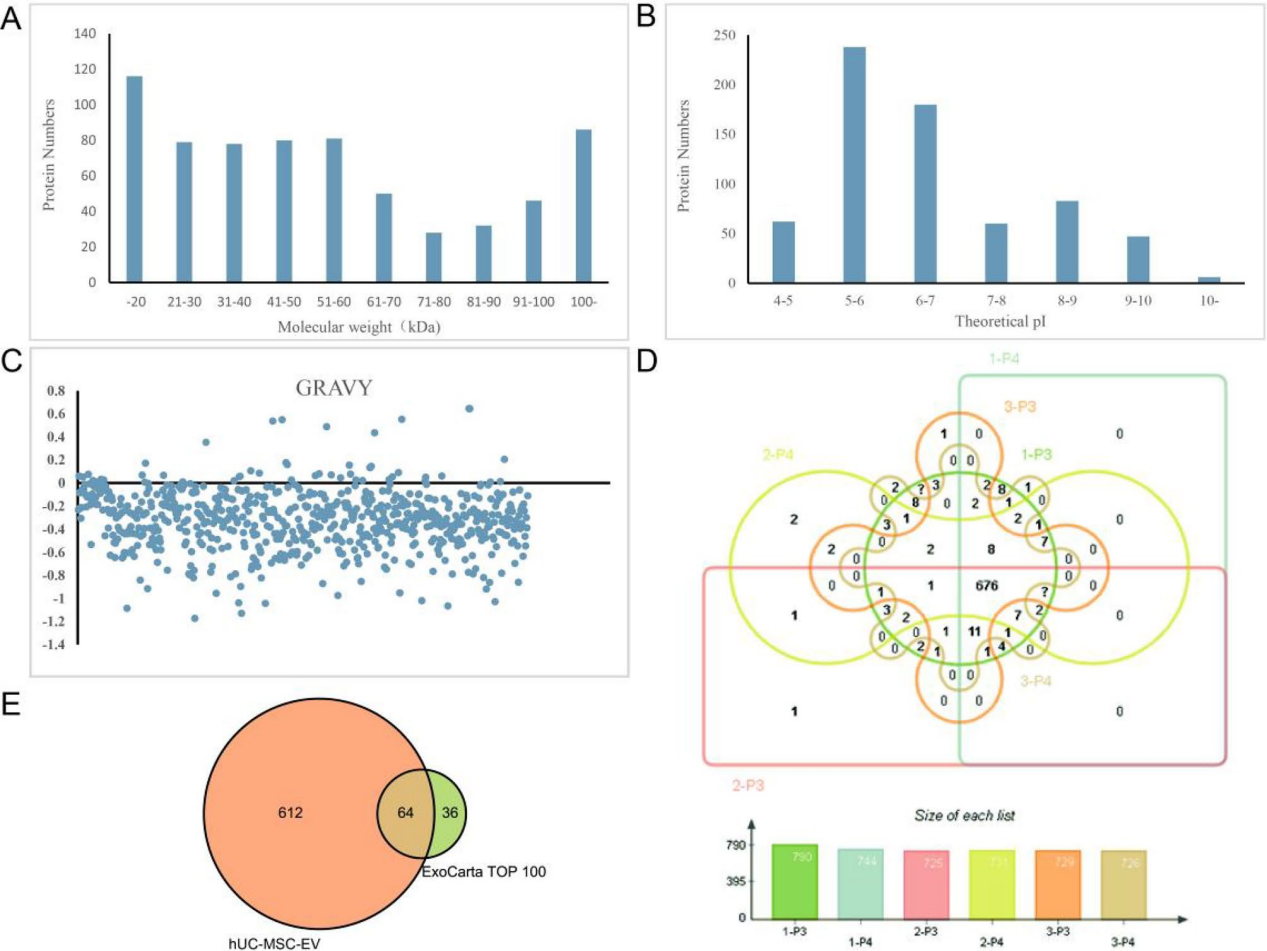
Evaluation of the hUC-MSC-EV proteome. (**A**) A molecular mass distribution map of the proteins is shown. (**B**) Statistical analysis of the protein isoelectric point distributions was performed. (**C**) The GRAVY values of the hUC-MSC-EV proteins varied from − 1.174 to 0.644, and 628 (92.9%) of them were negative (below zero). The proteins exhibiting positive GRAVY values were deemed hydrophobic, and those with negative GRAVY values were deemed hydrophilic. (**D**) The proteomic Venn diagram is shown. The proteomes of the third and fourth generations hUC-MSC-EVs from the three donors had 676 shared protein species. (**E**) Among the 676 proteins identified in EVs, 64 overlapped with the 100 most common EV marker proteins in the ExoCarta database.

A total of 807 unique proteins were identified by LC‒MS/MS in the third and fourth passages of hUC-MSC-EVs (1-P3, 1-P4, 2-P3, 2-P4, 3-P3, and 3-P4) from three donors. Analysis showed that the relative molecular weight and isoelectric point (pI) of the identified proteins were widely distributed (Fig. 4A,B), indicating that the performance of assay system was feasible. The GRAVY values identified in this study varied between − 1.174 and 0.644, among which 628 (92.9%) proteins were hydrophilic (Fig. 4C). The results indicated that most of the hUC-MSC-EV proteins detected by mass spectrometry were intramembrane proteins rather than membrane proteins. A Venn diagram was drawn for 790 proteins in 1-P3, 744 proteins in 1-P4, 725 proteins in 2-P3, 731 proteins in 2-P4, 729 proteins in 3-P3, and 726 proteins in 3-P4. The results showed that the six batches of hUC-MSC-EVs shared 676 unique protein species, and a small percentage of proteins were unique to a particular batch of hUC-MSC-EVs (Fig. 4D). The 676 proteins were compared with the top 100 most common EV marker proteins in the ExoCarta database, and 64 of the most common EV marker proteins were found (Fig. 4E). These results demonstrated the reliability of the analysis and stability of the hUC-MSC-EVs extracted in this study.

### Functional enrichment analysis of hUC-MSC-EVs proteome

**Figure 5:**
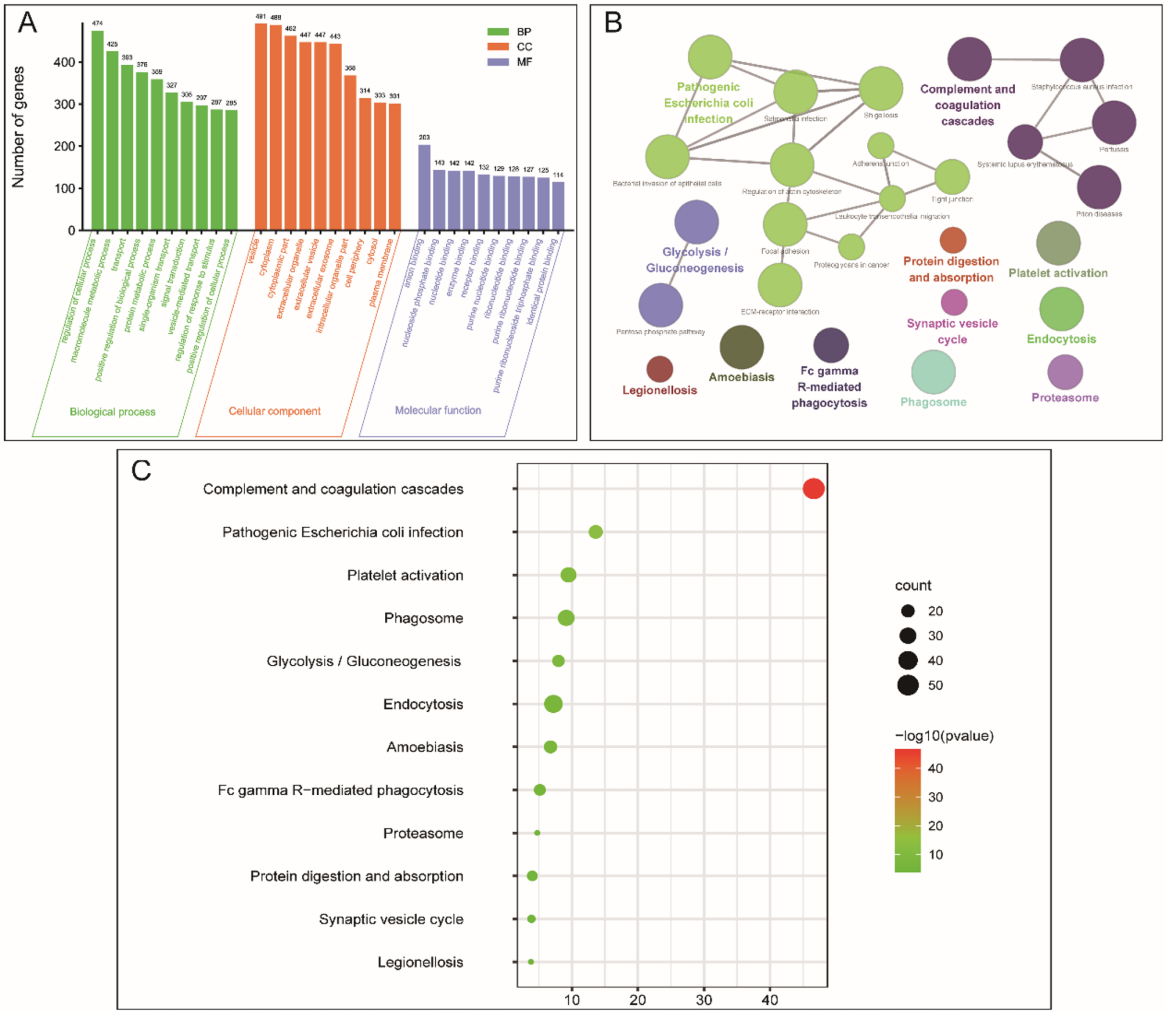
Bioinformatics analysis of the hUC-MSC-EV proteome. (**A**) GO analysis of the hUC-MSC-EV proteome, including BP, CC, and MF, was performed. (**B**) ClueGO network analysis of the hUC-MSC-EV proteome showed enrichment of effector proteins. (**C**) Bubble diagram of the KEGG enriched by the hUC-MSC-EV proteome (the ordinate is the name of the KEGG metabolic pathway, and the abscissa is the number of proteins annotated to the pathway and the proportion of the total number of proteins annotated).

We performed Gene Ontology (GO) function annotation of 676 hUC-MSC-EVs proteins and depicted the biological process (BP), cell component (CC), and molecular function (MF) terms. The results showed that the hUC-MSC-EV proteome was involved in molecular metabolism, protein metabolism, material transport, signal transduction, and other biological processes. These proteins were also enriched in the molecular functions receptor binding and binding to a variety of nucleotides. Furthermore, the hUC-MSC-EV proteome is also associated with the composition of various EVs and organelles. The top 10 level 2 GO annotations for BP, CC, and MF are shown in Fig. 5A. Additional KEGG analysis results are shown in Fig. 5B,C. The ClueGO plugin of Cytoscape was used for functional enrichment analysis (Fig. 5B). According to the above results, a bubble map of the enriched KEGG pathways of the hUC-MSC-EV proteome was generated (Fig. 5C). The hUC-MSC-EV proteome was shown to be associated with multiple regulatory pathways, including complement and coagulation cascades, pathogenic *Escherichia coli* infection, platelet activation, phagosome, glycolysis/gluconeogenesis, endocytosis, amoebiasis, Fc gamma R-mediated phagocytosis, proteasome, protein digestion and absorption, the synaptic vesicle cycle, and legionellosis. The synaptic vesicle cycle pathway has attracted much of our attention. In this pathway, synaptic vesicles (SVs) mediate communication between neurons by releasing neurotransmitters through repeated cycles of exocytosis and endocytosis. We hypothesized that hUC-MSC-EVs regulate neurotransmitter transmission through the synaptic vesicle pathway to treat neurological diseases. Therefore, hUC-MSC-EVs were used to treat APP/PS1 transgenic mice, with the hope of revealing the potential clinical application of hUC-MSC-EVs.

### Therapeutic potential of hUC-MSC-EVs for AD

**Figure 6:**
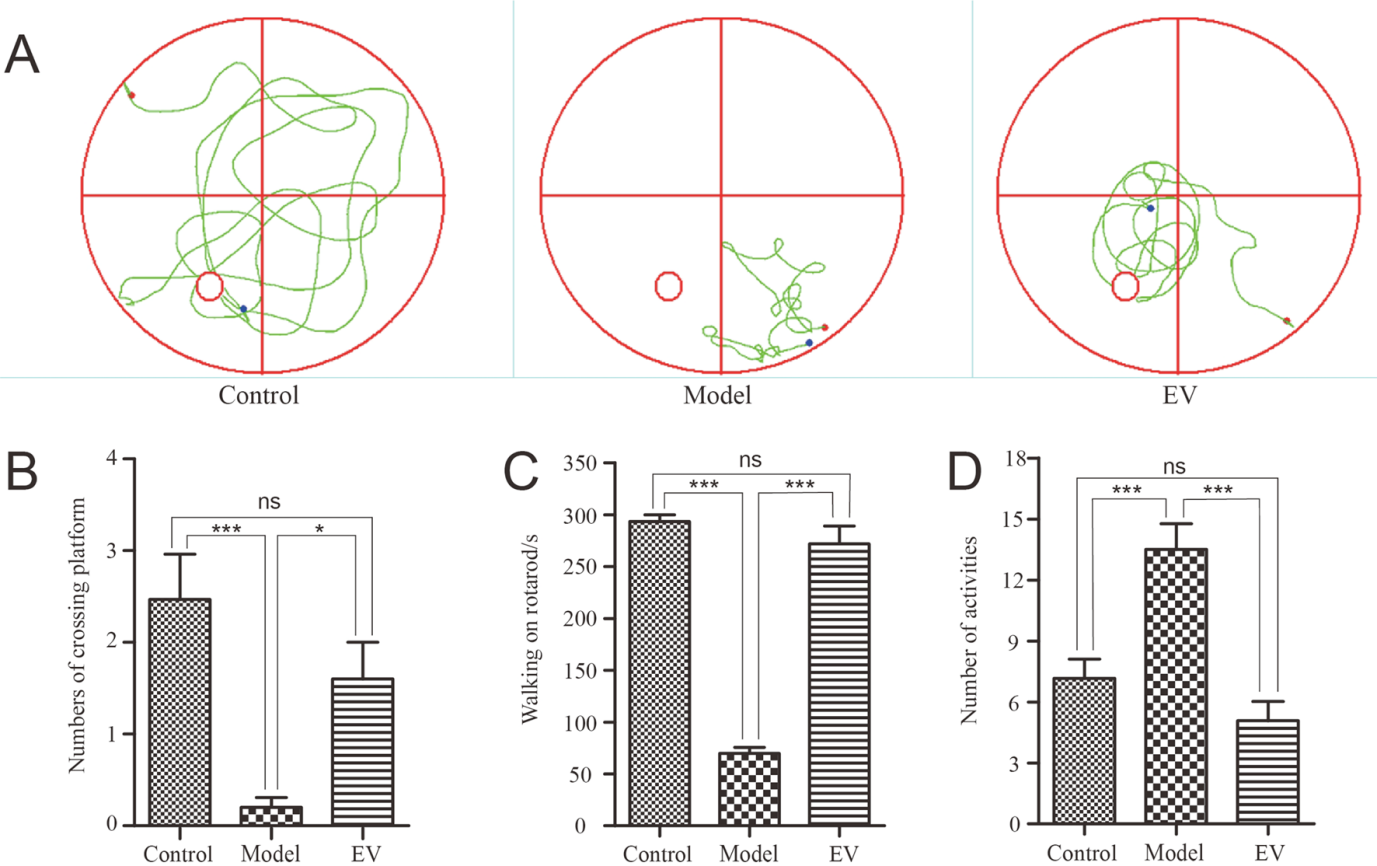
Effect of hUC-MSC-EVs on AD. (**A**) Movement of the mice in each group was evaluated using the Morris water maze test. The platform was found regularly in the control and EV groups but not in the model group. (**B**) The number of platform crossings in each group is shown. Mice in both the control and EV groups could find the platform multiple times within 1 min. (**C**) The time spent walking on the rotating rod was recorded. Mice in both the control and EV groups took significantly longer to walk on the rotating rod than did the model mice. (**D**) The number of mouse activities in the independent activity experiment in each group was compared. The number of voluntary active mice in the control and EV groups was significantly lower than that in the model group. The results are shown as the mean ± SEM (n = 6). *p < 0.05, ***p < 0.001, ns: no significant difference.

To investigate the therapeutic potential of hUC-MSC-EVs on AD, three experimental groups were utilized: the control (C57BL/6), model (APP/PS1), and EV (APP/PS1) groups. The EV-treated mice were treated with a tail vein infusion of hUC-MSC-EVs. As shown in Fig. 6A, Mice were trained with a platform for spatial learning and memory abilities, after training the platform was removed for further study, the resuls showed that the mice in the control and EV groups were mainly active in the interior of the pool, whereas mice in the model group were more active at the edge of the pool. Moreover, the model group exhibited limited activity in certain quadrants, while the other two groups exhibited traces of activity in all four quadrants. The number of platform crossings was statistically analysed (Fig. 6B), and the average number times of platform crossings was significantly lower in the model group than in the control and EV groups(p < 0.05). These results suggested that spatial learning and memory abilities are impaired in APP/PS1 mice and improved after hUC-MSC-EV intervention. In addition, the rotarod test and autonomous experiments were also performed to evaluate the exercise ability, fatigue, and despair of mice. As shown in Fig. 6C,D, the walking time on the rotating rod in the control group and the EV group was significantly longer than that in the model group, but the number of spontaneous activities in the control group and the EV group was significantly lower than that in the model group (p < 0.001). The above results showed that, compared with those in the control group, the exercise ability of the model group significantly decreased and there was a significant increase in fatigue. Emotion was more uncontrollable and improved after treatment with hUC-MSC-EVs. Therefore, hUC-MSC-EVs have therapeutic potential for treating AD.

### hUC-MSC-EVs interfere with AD through the synaptic vesicle cycle

**Figure 7:**
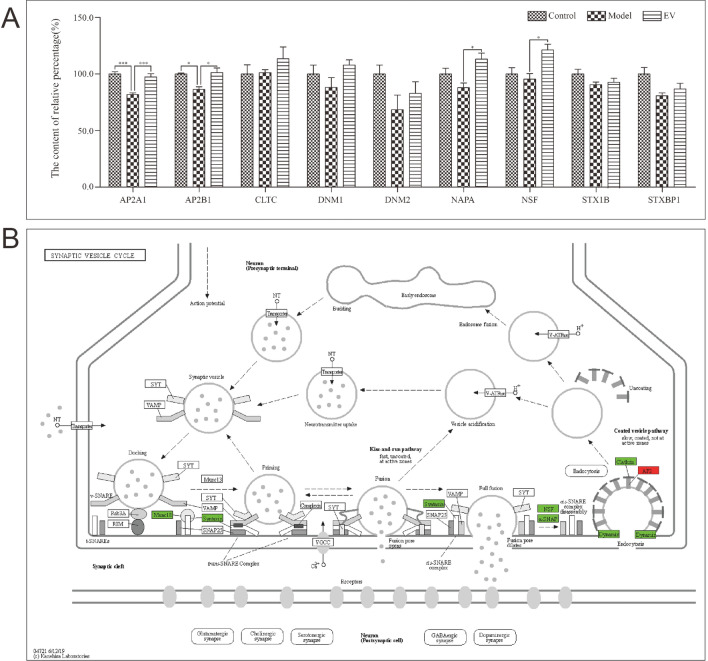
Changes in the protein content in the hippocampus of mice in different groups. (**A**) The percentage of histone content in the other two groups was calculated when the histone content of the control was 100%. The results showed that AP2A1 and AP2B1 levels in the hippocampus of the model group were significantly lower than those in the control group and EV groups. NAPA and NSF levels in the hippocampus of the EV group were significantly greater than those in the model group. Moreover, there was no significant difference between the other groups. The results are shown as the mean ± SEM (n = 3). *p < 0.05, ***p < 0.001. (**B**) A synaptic vesicle cycle diagram (https://www.genome.jp/pathway/map04721)^24^ is shown. The nine proteins in the hUC-MSC-EV proteome that were enriched in the synaptic vesicle cycle pathway (AP2, AP2A1 and AP2B1; Dynamin, DNM1 and DNM2) are highlighted in colour. In the hippocampus of mice treated with hUC-MSC-EVs, the levels of the green-labelled proteins did not significantly change compared with those in the control group. In contrast, there were significant changes in the red-labelled proteins in the control and EV groups compared with those in the model group after hUC-MSC-EV treatment. The figure was prepared using normal tools only and not used any specialized tool.

To explore whether hUC-MSC-EV proteins can interfere with AD through the synaptic vesicle cycle, we investigated nine proteins in the hUC-MSC-EVs proteome that were found to be enriched in the synaptic vesicle cycle pathway. LC‒MS/MS was subsequently used to determine the relative levels of the above mentioned proteins in the hippocampus of the different groups of mice. The results of LC‒MS/MS shown in Fig. 7A indicate that the levels of AP2A1 and AP2B1 in the hippocampus of the model group were significantly lower than those in the control group and the EVs group (p < 0.05). Similarly, the levels of NAPA and NSF in the hippocampus of the EV group were significantly greater than those in the model group, but there was no difference between the control group and the model group. By searching for the above nine proteins in the synaptic vesicle cycle pathway downloaded from KEGG (https://www.genome.jp/pathway/map04721)^24^, we found that AP2A1 and AP2B1 are related to the endocytosis of vesicles (Fig. 7B).

## Discussion

In the past decade, stem cell technology, as an integral part of regenerative medicine, has shown great potential. Compared with other stem cells, MSCs have obvious advantages in the treatment of clinical diseases because of their multiple differentiation, self-renewal, homing, and immunomodulatory abilities^25–27^. Due to non-invasive source recovery, hUC-MSCs exhibit no ethical problems, low immunogenicity, rapid self-renewal ability, stable doubling time, and high proliferative efficacy; thus, they are widely used in stem cell therapy and regenerative medicine^28,29^. However, stem cell therapy still has several limitations. Unstable biological activity, over-differentiation, and tumorigenicity are among the problems associated with these agents; thus, researchers hope to find better alternatives. Increasing evidence shows that the therapeutic effect of MSCs is mediated mainly by extracellular vesicles, especially the paracrine effects of EVs, which mainly function to facilitate microenvironment repair and immune regulation^30,31^. Compared to MSCs, the advantages of EVs include their biocompatibility, low immunogenicity, and no vascular occlusion tendencies, which make them unique in clinical treatment.

In this study, EVs were enriched and purified from hUC-MSCs culture media from different donors, the proteomic of EVs were characterized by LC‒MS/MS. Several studies have shown that EVs can alleviate oxidative damage and promote tissue repair^32–34^. Notably, these studies have focused more on noncoding RNAs, such as miRNAs, in EVs. Since proteins are the most direct determinant of function, their roles are worthy investigated. Although there are proteome studies on EVs derived from MSCs, there are few studies on the proteome of different batches of hUC-MSC-EVs.

To analyse the related information about the proteomes of different batches of hUC-MSC-EVs, two generations of hUC-MSC-EVs were identified from three different donors. By LC–MS/MS, a total of 807 kinds of non-redundant proteins were identified. Among them, 676 kinds of proteins were found in the 6 batches of hUC-MSC-EVs. Sixty-four of these proteins were found in the ExoCarta TOP 100 list, indicating that the hUC-MSC-EVs proteome exhibits similarities to that of EVs.

Additionally, the biological function of the identified hUC-MSC-EVs was revealed by GO analysis. The identified proteins were involved in 88 molecular functions, the most important of which included cellular and molecular binding, such as anion binding, nucleotide binding, receptor binding, and ribonucleotide binding. EVs can bind to different cells through these molecular binding functions, thus activating the functions of cell membrane fusion and receptor cell membrane proteins^35^. Moreover, the hUC-MSC-EVs also participated in 648 biological processes, which involved the regulation of cellular processes, macromolecule metabolic processes, and transport processes. The hUC-MSC-EV proteins were also enriched in 157 cellular components. The most enriched cell component was in the synaptic vesicle cycle signalling pathway.

To further explore the possible signalling pathways regulated by the hUC-MSC-EV proteome, KEGG pathway analysis was performed. These proteins were involved in the regulation of blood coagulation, bacterial infection, and phagocytosis, as well as vesicle circulation. The above bioinformatics analysis revealed the potential neuroregulatory effect of hUC-MSC-EVs. Among the KEGG pathways related to neuro regulation, the most noteworthy was the synaptic vesicle cycle signalling pathway. In this signalling pathway, SVs near nerve endings actively transport neurotransmitters through repeated exocytosis and endocytosis cycles. SVs loaded with neurotransmitters dock in a special region of the presynaptic plasma membrane called the active region, where they initiate responses. Once the action potential arrives, Ca^2+^ enters through a voltage-gated channel, and neurotransmitters are released through exocytosis, usually in less than a millisecond. After fusion, SVs are withdrawn by endocytosis and reloaded for another round of exocytosis^36^. Based on the above information, we speculated that the hUC-MSC-EV proteome may regulate the transport of neurotransmitters through the synaptic vesicle cycle signalling pathway and play a role in neural information transmission.

To verify the above conjecture, hUC-MSC-EVs were used to treat AD model APP/PS1 transgenic mice. The results of behavioural experiments showed that hUC-MSC-EV intervention significantly enhanced the spatial memory, learning ability, and exercise ability and decreased fatigue in APP/PS1 mice. The relative protein concentration in the hippocampus of the mice was also analysed via LC‒MS/MS. The results showed that the hUC-MSC-EV proteome was enriched in nine proteins found in the synaptic vesicle cycle signalling pathway, including AP2A1, AP2B1, clathrin heavy chain (CLTC), dynamin-1 (DNM1), dynamin-2 (DNM2), alpha-soluble NSF attachment protein (NAPA), vesicle-fusing ATPase (NSF), syntaxin-1B (STX1B), and syntaxin-binding protein 1 (STXBP1). CLTC is a major protein component of the cytoplasmic face of intracellular organelles, including coated vesicles and coated pits. These specialized organelles are involved in the intracellular trafficking of receptors and endocytosis of a variety of macromolecules. Furthermore, CLTC may be a key regulatory gene in the prefrontal cortex of AD patients^37^. DNM1 and DNM2 are the subfamilies of GTP-binding proteins that are involved in clathrin-mediated endocytosis and other vesicular trafficking processes. They are also closely linked to the development of both Aβ and tau pathologies^38,39^. NSF is a vesicular ATPase that is essential for AMPA-type glutamate receptor (AMPAR) trafficking. Research has shown that tau interacts with and dose-dependently reduces the activity of NSF^40^. NAPA plays a role in the completion of membrane fusion by mediating the interaction of NSF with the vesicle-associated and membrane-associated SNAP receptor (SNARE) complex, and stimulating the ATPase activity of NSF. STX1B belongs to a family of proteins thought to play a role in the exocytosis of synaptic vesicles. Vesicle exocytosis releases vesicular contents and is important for various cellular functions^41^. STXBP1 is a protein that plays a critical role in presynaptic vesicle release, and its mutation is closely associated with neurodevelopmental disorders^42^. Nevertheless, the exact role of STXBP1 in AD has not been determined.

The protein levels of AP2A1 and AP2B1 decreased significantly in the hippocampus of APP/PS1 mice. After intervention with hUC-MSC-EVs, the levels of AP2A1 and AP2B1 normalized. By tagging the synaptic vesicle cycle pathway, we found that AP2A1 and AP2B1 are involved in endocytosis. AP2A1 and AP2B1 encode the alpha 1 adaptin subunit of the adaptor protein 2 (AP-2) complex that are found in clathrin-coated vesicles. The AP-2 complex is a heterotetramer consisting of two large adaptins (alpha or beta), a medium adaptin (mu), and a small adaptin (sigma)^43^. AP-2 is involved in clathrin-dependent endocytosis in which cargo proteins are incorporated into vesicles surrounded by clathrin (clathrin-coated vesicles, CCVs), which are destined for fusion with early endosomes. The clathrin lattice serves as a mechanical scaffold but is itself unable to bind directly to membrane components^44^. Clathrin-associated adaptor protein (AP) complexes, which can bind directly to both the clathrin lattice and the lipid and protein components of membranes, are considered to be the major clathrin adaptors involved in CCV formation. AP-2 also serves as a cargo receptor to selectively sort the membrane proteins involved in receptor-mediated endocytosis^45^. AP-2 seems to play a role in recycling synaptic vesicle membranes from the presynaptic surface^46^. During long-term potentiation in hippocampal neurons, AP-2 is responsible for the endocytosis of ADAM10^47^. The above results confirmed that hUC-MSC-EVs can regulate AP2A1 and AP2B1 through the synaptic vesicle cycle signalling pathway, thus affecting the endocytic effect of SVs and alleviating AD. The available data so far warrants further investigation of how hUC-MSC-EVs treatment correlates to the changes of AP2A1 and AP2B1 at transcription and translation levels in vitro and in vivo; It is also important to conduct gene over-expression or knockdown studies of the relevant signalling components to verify whether AP2A1 and AP2B1 regulate the synaptic vesicular circulation signalling pathway and affect the long-term enhancement of hippocampus neurons. Furthermore, it is critical to find out if AP2A1 and AP2B1 are co-located with Aβ pathology or Tau pathology to further regulate the pathophysiological process of AD formation and development. We will evaluate if AP2A1 and AP2B1 can be used as novel biological diagnostic markers to provide new ideas for the clinical diagnosis and treatment of AD.

In conclusion, the proteins in the second generation of hUC-MSCs from three different donors were assessed via LC‒MS /MS, and the 676 proteins identified in the hUC-MSC-EVs from the six batches were identified as the core proteome of hUC-MSC-EVs. Through bioinformatics analysis of the hUC-MSC-EV core proteome, potential therapeutic efficacy of these proteins against nervous system diseases was found. A possible therapeutic mechanism was found by verifying the effects in an AD mouse model. These results provide a basis for further exploration of the potential of hUC-MSC-EVs as therapeutic agents.

## Conclusions

The above results revealed that the protein composition of human umbilical cord mesenchymal EVs in different batches was basically the same. The specific mechanism their alleviation of AD through the synaptic vesicle cycle pathway should be further discussed. The results of this study can provide a theoretical basis for the use of human umbilical cord mesenchymal stem cell-derived EVs to treat diseases and further promote their clinical application.

## Methods

### Stable passage and identification of hUC-MSCs

Primary hUC-MSCs were obtained from Jianyuan Precision Medicine (Zhangjiakou) Co., Ltd. All the donors provided informed consent. All procedures were approved by the ethics committee. Three samples of cells from different donor sources were digested for 2 min at room temperature with 0.25% trypsin until 80% of the cells were detached and floated, after which the cell suspension was terminated with culture media. The terminated cell suspension was collected in a centrifuge tube and centrifuged at 1000 rpm for 10 min. Next, 2 × 10^6^ cells were transferred to a new T175 culture vial, maintained in complete MEM (HyClone, China) supplemented with 5% EliteGro-Adv (EliteCell, China) and incubated at 37 °C and 5% CO_2_. The culture flask was observed under a microscope every day and passaged when the cell confluence reached 80–90%. The cells from 3 different donors were stably passaged to the third (P3) or fourth generation (P4). Cell morphology was observed under an inverted microscope. The cell surface markers CD73+, CD90+, CD105+ ≥ 95%, CD45+, and CD34+ ≤ 5% were analysed by flow cytometry. Cell subculture was stopped at this time.

### Extraction and identification of hUC-MSC-EVs

In this study, EVs were enriched from typical hUC-MSCs culture with EV-free and serum-free media. Specifically, the third and fourth generations of hUC-MSCs were cultured in serum-free medium (5% EliteGro-Adv + 95% MEM) until the fusion rate reached to 90%. After washing with PBS twice, the fresh serum-free medium was added and the samples were incubated for 48 h. The culture supernatant was collected under aseptic condition, followed by centrifugation at 300×*g* for 10 min to remove dead cells. The resulting culture supernatant was subject to further centrifugation at 2000×*g* for 10 min using an F15-8 × 50cy rotor (Thermo, USA) and the resulting supernatant was filtered with 0.22-μm membrane filter (Navigator, China). After filtration, the supernatant was subject to ultracentrifugation at 4 °C and 100,000×*g* for 70 min using a P100AT2 rotor (Himac, Japan). The resulting pellets were further re-suspended with pre-cooled PBS, followed by ultracentrifugation at the same parameters. The final pellets were collected and re-suspended with pre-cooled PBS for the next analysis. Transmission electron microscopy (Hitachi, Japan) was used to detect the morphology of EVs. The EVs (1 μg/10 μl PBS) were dropped onto the sample copper net and let stand at room temperature for 2 min. The liquid was removed from the side of the filter with filter paper, and then negatively stained with 10 μL of 2% uranyl acetate solution at room temperature for 1 min. The negative control dye was absorbed by filter paper, after which the mixture was dried at room temperature. After natural drying, the samples were examined using transmission electron microscopy (Hitachi, Japan) at 60 kV. The particle size and concentration of the EVs were determined using a Zetasizer Lab nanometre particle size and Zeta potential analyser (Malvern Panalytical, UK). The surface markers of EVs, CD81, and CD63, were analysed by flow cytometry. Western blot was used to assess the presence of negative EV markers.

### LC‒MS/MS analysis of the proteome

#### Extraction and preparation of EV proteins and hippocampal tissue proteins

The exudates of 1-P3, 1-P4, 2-P3, 2-P4, 3-P3, and 3-P4 from the hUC-MSC-EVs were extracted. Then, 50 μL was added to 2% SDS, 4,300 μL was added for ultrasonic comminution, and the samples were centrifuged at 8000×*g*. The supernatant was collected.

Mice were anaesthetized with sodium pentobarbital. The chest was opened from the xiphoid process with scissors to expose the heart's position. The needle of the infusion device punctured the apex of the heart at a depth of approximately 4 mm. The needle was fixed with tweezers, and the right atrial appendage was cut. The blood vessels were washed with normal saline until there was no blood outflow from the right atrial appendage. The hippocampal tissue was separated, stored in a frozen tube and frozen in liquid nitrogen. The hippocampal tissue of the mice was fully ground in a homogenizer with PBS and protease inhibitor. The grinding solution was stored at 4 °C and the samples were centrifuged at 12,000×*g* for 5 min, after which the supernatant was collected. All the sacrifice procedures were performed under sodium pentobarbital anaesthesia, and efforts were made to minimize animal suffering.

The same volume of Tris-saturated phenol was added to the collected hUC-MSC-EVs and mouse hippocampal supernatant. After mixing, the solution was centrifuged at 12,000×*g* for 5 min to achieve stratification. The upper aqueous phase was removed while keeping the liquid organic phase intact. The same volume of 50 mM Tris–HCl was added, and the solution was mixed well. The mixture was stored at 4 °C and centrifuged at 12,000×*g* for 15 min. The above centrifugation steps were subsequently repeated. Five times the volume of precooled ammonium acetate methanol was added to the retained organic phase, the mixture mixed well, and the mixture was precipitated overnight at − 20 °C. The solution was centrifuged under the same conditions, after which the precipitate was retained. One millilitre of methanol was added to the precipitate, and the sample was mixed well and transferred to a 1.5 mL EP tube. This solution was centrifuged under the same conditions, and the supernatant was discarded. This step was repeated twice. Afterwards, 10 μL of DTT was added to the precipitated protein sample for 30 min at 37 °C. IAA (20 μL) was subsequently added to the precipitated protein sample, after which the mixture was protected from light at 26 °C for 30 min. Then, 500 μL of trypsin was added at a ratio of 1:50 (v:v) at the end of reductive alkylation, after which the mixture was hydrolysed at 37 °C for 10 h. The above enzymatically hydrolysed peptides were desalted via solid-phase extraction with C18 SPE (Anpu, China), after which the mixture was dried and set aside.

#### LC‒MS/MS analysis

The polypeptide samples were redissolved in pure water (Fisher, USA) containing 0.1% formic acid, and the iRT reagent (Biognosys, Switzerland) was added to prepare for subsequent MS identification. The polypeptide samples were separated by UPLC (Mtel Classwork Waters, USA). The separation was carried out on a C18 reversed-phase chromatographic column (1.8 μm particle size, 75 μm ID × 250 mm length; Waters, USA). Phase A was pure water (Fisher, USA) containing 0.1% formic acid, and phase B was acetonitrile (Fisher, USA) containing 0.1% formic acid. The elution flow rate was 300 nL/min, and the elution gradient was mobile phase B from 2% Mel to 35% for 2 h. The polypeptide samples were sprayed into Q Exactive HF mass spectrometer (Thermo, USA) via an ion source for DIA-based quantitative analysis. The MS parameters were set as follows: (A) The DIA mode was based. The full scanning range was set at 350 to 1200 m/z. The scanning resolution of the parent ion was set to 60,000. The AGC was set to 3e6, and the maximum ion implantation time was set to 50 ms. (B) HCD was used for fragmentation, and the collision energy was set to 27%. (C) The DIA method was set as follows: full MS (350 to 1250 m/z), followed by 20 DIA MSMS. The DIA isolation window (IW) was set at 59.0 m/z, 25.0 m/z, 19.0 m/z, 17.0 m/z, 13.0 m/z, 13.0 m/z, 11.0 m/z, 12.0 m/z, 12.0 m/z, 11.0 m/z, 12.0 m/z, 9.0 m/z, 10.0 m/z, 10.0 m/z, 11.0 m/z, 10.0 m/z, 10.0 m/z, 9.0 m/z, 10.0 m/z, and 10.0 m/z; then Full MS (350–1250 m/z), followed by 20 DIA MSMS (IW) at 10.0 m/z, 9.0 m/z, 10.0 m/z, 8.0 m/z, 9.0 m/z, 9.0 m/z, 10.0 m/z, 10.0 m/z, 10.0 m/z, 10.0 m/z, 9.0 m/z, 10.0 m/z, 10.0 m/z, 10.0 m/z, 10.0 m/z, 10.0 m/z, 11.0 m/z, 10 m/z, 10 m/z, and 11 m/z; then, full MS (350–1250 m/z), followed by 20 DIA MSMS (IW) at 10 m/z, 11 m/z, 12 m/z, 11 m/z, 13 m/z, 13 m/z, 13 m/z, 14 m/z, 13 m/z, 14 m/z, 14 m/z, 19 m/z, 18 m/z, 20 m/z, 27 m/z, 24 m/z, 33 m/z, 45 m/z, 56 m/z, and 91 m/z. (D) The MS2 scan resolution was set to 30,000 and the AGC target was set to 1e6 ^23^.

#### Database searching and bioinformatics analysis

The original data were analysed with Spectronaut software version 15.0 (Biognosys AG, Schlieren, Switzerland) and standard DIA analysis (FDR < 1%). The database of mouse proteins (UniProt, download date, protein quantity) was used to search for DIA. To rule out the possibility of contamination in the results, the human keratin sequence was used as the contaminated database for proteomic searching. The selection of the search parameters was as follows: (A) digestion at two misfolded sites; (B) variable modifications adjusting the oxidation of methionine; and (C) fixed changes adjusting cysteine carbamidomylation. The proteomics data from mass spectrometry were stored in the ProteomeXchange Consortium (http://proteomecentral.proteomexchange.org) using the iProX partner repository with the dataset identifier PXD037877 ^23^.

#### Data analysis

To screen the retrieved data, unique peptides ≥ 2, a Q value < 0.05, and a CV < 20% were selected as parameters for further analysis. The UniProt database (https://www.uniprot.org/) searches proteins based on molecular weight, isoelectric point (PI), and the ExPASy (ProtParam section of https://www.ExPASy.org/) website (https://www.ExPASy.org/resources/protparam). These data were used to calculate the grand average of hydropathicity (GRAVY) for each protein. Six groups of EV proteins were subsequently analysed with a Venn diagram (http://bioinformatics.psb.ugent.be/webtooLs/Venn/). The TOP100 EV marker proteins were downloaded from the ExoCarta database (http://www.exocarta.org/).

#### Bioinformatics analysis

Functional enrichment analysis of the hUC-MSC-EV proteome was carried out on the official GO website (http://www.exocarta.org/), and the BP, CC, and MF terms were evaluated. The KEGG website (https://www.kegg.jp/kegg/) was used to analyse the enrichment of the protein signalling pathways.

### Grouping and administration to mice

All the mice were purchased from Beijing Huafukang Biotechnology Co., Ltd., and all the animal experiments were conducted in accordance with ethical standards. All mice were provided free access to food and water and were kept in a colony room under a 12-h dark–light cycle in the Hebei Normal University Animal Center. All the animal procedures were performed in strict conformity with the Guidelines for Care and Use of Laboratory Animals of Hebei Normal University, approved by the Science and Technology Ethics Committee of Hebei Normal University, and complied with the ARRIVE guidelines. All methods were performed in accordance with the relevant guidelines and regulations. Twelve 3-month-old male APP swe/PS1dE9 double transgenic mice were randomly divided into a model group and an EV group of 6 animals each, and 6 litters of the same male mice with a C57BL/6J background were used as a control group; the mice weighed 25–30 g each. The protein concentrations of the six batches of hUC-MSC-EVs that were determined by the BCA assay kit were 10, 9, 9.5, 8, 9, and 10 μg/μL. Six batches of EVs were mixed and diluted to 2.5 μg/μL for subsequent animal experiments. The mice in the EV group were injected with 40 μL of hUC-MSC-EVs, and the mice in the model and control groups were given the same volume of PBS. All the mice were injected every 5 days for a total of 6 injections.

### Behavioural experiments in mice

#### The Morris Water Maze experiment

This experiment was designed to examine spatial learning and memory in mice by allowing them to swim to find a platform hidden in water. The Morris water maze is composed of a pool with a diameter of 120 cm and a depth of 50 cm, a platform, and a video analysis system. The water temperature was held at a constant (24 ± 2) °C, and the samples were surrounded by curtains to prevent external interference. The experiment was divided into a positioning navigation experiment and a space exploration experiment. The first 6 days were used for the positioning navigation experiment. Mice were put into the water with their back to the pool wall in each quadrant every day. The experiment ended automatically if the mouse found the platform and remained there for 10 s. If the platform was not found in the first 60 s, the mouse was guided to the platform in the next 30 s. On the 7th day, the space exploration experiment was conducted. The platform was removed, and the mice entered the water from any quadrant. The swimming paths within the first 60 s were recorded, and the number of times the mice crossed the platform was counted.

#### Rotarod test

In this study, a YSL-4C rotary-type fatigue metre was used for testing. The maximum rotation speed of the rotary-type fatigue metre was 40 revolutions per minute (RPM), and the maximum test time was 5 min. Behavioural experiments were conducted in a quiet environment, so the training was conducted and tested in a special behavioural laboratory. In the first two days, training occurred three times a day. The rotation speed was set to 40 RPM, and the rotation time was set to 5 min. At the beginning of training, the mice often fell off the rotary metre as the speed of the rotary metre increased. At this time, the mice were returned to the corresponding track and allowed to continue moving until the end of their 5 min of training. On the third day, the formal experiment was carried out, and each mouse was tested three additional times. Twenty minutes were left between each experiment for the mice to rest. The number of times the mice were on the rod spinner before they fell was recorded, and the number of mice that did not fall in the first 5 min was recorded, and the experiment was ended. The average of the three tests provided the daily test results.

#### Voluntary activity experiment

The mice were placed in the reaction tank of the ZZ-6 mouse autonomous activity tester, and the lid was closed for 2 min after adaptation. The activity times of the mice in the first 5 min were recorded. When the mice were active, the infrared probe under the reaction tank was used to detect the signal change, and the data were automatically recorded and displayed on the display screen. Each experiment required cleaning of the box to minimize the influence of the previous mouse on the next mouse.

### Statistical analysis

Statistical analysis was performed using SPSS 25.0 and Prism 5. The statistical significance (p < 0.05) was determined using Student's t test. The data are presented as the mean ± SEM.

### Ethics approval

All the animal procedures were performed following the Guidelines for Care and Use of Laboratory Animals of Hebei Normal University and approved by the Science and Technology Ethics Committee of Hebei Normal University (2023LLSC037).

## Data Availability

The proteomics data frommass spectrometry was stored in the ProteomeXchange Consortium (http://proteomecentral.proteomexchange.org) through the iProX partner repository with the dataset identifier PXD037877.
